# A High-Throughput Platform for Lentiviral Overexpression Screening of the Human ORFeome

**DOI:** 10.1371/journal.pone.0020057

**Published:** 2011-05-24

**Authors:** Dubravka Škalamera, Max V. Ranall, Benjamin M. Wilson, Paul Leo, Amy S. Purdon, Carolyn Hyde, Ehsan Nourbakhsh, Sean M. Grimmond, Simon C. Barry, Brian Gabrielli, Thomas J. Gonda

**Affiliations:** 1 University of Queensland Diamantina Institute, Princess Alexandra Hospital, Brisbane, Queensland, Australia; 2 Institute for Molecular Bioscience, University of Queensland, St. Lucia, Queensland, Australia; 3 Molecular Immunology Laboratory, Discipline of Paediatrics, University of Adelaide and Women's and Children's Health Research Institute, Women's and Children's Hospital, North Adelaide, South Australia, Australia; National Institutes of Health, United States of America

## Abstract

In response to the growing need for functional analysis of the human genome, we have developed a platform for high-throughput functional screening of genes overexpressed from lentiviral vectors. Protein-coding human open reading frames (ORFs) from the Mammalian Gene Collection were transferred into lentiviral expression vector using the highly efficient Gateway recombination cloning. Target ORFs were inserted into the vector downstream of a constitutive promoter and upstream of an IRES controlled GFP reporter, so that their transfection, transduction and expression could be monitored by fluorescence. The expression plasmids and viral packaging plasmids were combined and transfected into 293T cells to produce virus, which was then used to transduce the screening cell line. We have optimised the transfection and transduction procedures so that they can be performed using robotic liquid handling systems in arrayed 96-well microplate, one-gene-per-well format, without the need to concentrate the viral supernatant. Since lentiviruses can infect both dividing and non-dividing cells, this system can be used to overexpress human ORFs in a broad spectrum of experimental contexts. We tested the platform in a 1990 gene pilot screen for genes that can increase proliferation of the non-tumorigenic mammary epithelial cell line MCF-10A after removal of growth factors. Transduced cells were labelled with the nucleoside analogue 5-ethynyl-2′-deoxyuridine (EdU) to detect cells progressing through S phase. Hits were identified using high-content imaging and statistical analysis and confirmed with vectors using two different promoters (CMV and EF1α). The screen demonstrates the reliability, versatility and utility of our screening platform, and identifies novel cell cycle/proliferative activities for a number of genes.

## Introduction

Elucidating gene function is a pressing challenge for human biology and medicine. Given that the human genome consists of up to 25000 protein-coding genes [1], this task requires high-throughput approaches. An additional requirement of such approaches is flexibility of the research platform, since each gene can participate in multiple functional networks depending on biological and environmental conditions.

Although gene function can be inferred from sequence homology to characterised genes as well as expression patterns, the most definitive answers come from observing how altering expression of a gene affects phenotype. Reducing or completely abolishing gene expression by gene silencing can identify genes that are necessary for a particular cellular function, while induced overexpression points to genes that are sufficient to generate a phenotype. In addition, overexpression allows for analysis of subcellular protein localisation as well as *in-vivo* protein-protein interactions. High-throughput technology for gene silencing through siRNA, and to a lesser extent shRNA, has been developed and is now extensively used to screen the human genome [2], [3]. In contrast, so far only a few studies have investigated the effect of ectopic cDNA expression on a genomic scale using individually arrayed expression clones. This is in part due to the fact that gene silencing can be achieved with readily synthetised oligonucleotides while overexpression requires cloning full length open reading frames (ORFs) into expression plasmids [4], [5]. Another difficulty is that foreign plasmids can be easily transfected into only a limited number of human cell types, so that the existing reports have focused on highly transfectable cell lines such as HEK293T [6], [7], [8], U2OS2 [9], HCT116 [10] and SMC1772 [11]. Here we describe a high-throughput platform for overexpression screening of the human genome in an arrayed one gene per well format that circumvents these difficulties by using Gateway cloning and lentiviral expression vectors.

Lentiviral vectors deliver genes into chromosomes of both dividing and non-dividing cells, allowing stable expression of transgenes even in cell lines refractory to transfection [12]. The range of screenable cell types is further increased by using viral packaging vectors with pan-tropic envelope proteins such as VSV-G, which allows transduction of most mammalian cell types. Once the viral supernatant is generated, it can be used on multiple cell lines simultaneously, adding another level of flexibility to viral vector-based screening platforms.

A number of collections of human ORFs derived by PCR from the Mammalian Gene Collection have become available in Gateway-compatible entry vectors [13], [14], [15], allowing for shuffling of the ORFs between vectors with efficiency and scalability greatly exceeding that of traditional cloning methods [4], [16], [17]. We have employed a robotic liquid handling system to optimise Gateway cloning in 96-well microplate format and used it to transfer human ORFs from the hORFeome collection [15] into a lentiviral expression plasmid. Using the same microplate format, we have devised a protocol for robotic transfection of the HEK293Tcell line which was used to generate an arrayed library of viral supernatants ready for screening. To test the platform, we performed a pilot screen using a high-content imaging assay for cell-proliferation.

Cell proliferation control is an essential requirement for all multicellular organisms and is dependent on complex, highly organized gene interaction networks. Although cell-cycle regulation has been extensively studied and is well understood in a number of species, many key components remain elusive. This is particularly evident in cancer, where the diseased state is generated in part by the cells escaping normal proliferation control [18], [19]. As the number of genetic perturbations observed in cancer grows into thousands, it becomes increasingly difficult to determine which of these changes are driving the disease process and would therefore make suitable targets for anti-cancer therapy [20]. Putative candidates could be identified in a genetic overexpression screen for genes that can drive abnormal cell proliferation. Since changes in cell proliferation rate can be caused by variety of external stimuli, methodologies developed for this screen are applicable to analysis of other cellular functions such as response to pathogens, toxins, nutrients or drugs.

Here we describe a screen for genes that could induce cells to proliferate in the absence of necessary growth factors. We used arrayed viral supernatants to overexpress 1990 genes in the non-tumorigenic human mammary epithelial cell line MCF-10A, and looked for genes that promote cell proliferation after removal of the epidermal growth factor (EGF), which is required for continued growth in this cell line. In order to keep the platform scalable to whole genome investigation, we used a previously described end-point DNA synthesis assay as a marker for proliferation [21]. The assay relies on quantifying nucleotide analogue EdU incorporation in transduced and untransduced cells in each well, using a high-content imager. Putative hits were subsequently confirmed in time course experiments involving two different expression vectors.

## Results

### Library construction

For this pilot study we picked 1320 genes for which published expression profiles or functional screening data suggested involvement in cell cycle regulation [22], [23], [24], [25], myeloid cell proliferation [26] and/or protein phosphorylation [27], [28], as well as 670 randomly selected clones (Table S1). Predicted insert size ranged from 84 to 7116 bp, ensuring that developed protocols were not biased for particular ORF length.

**Figure 1:**
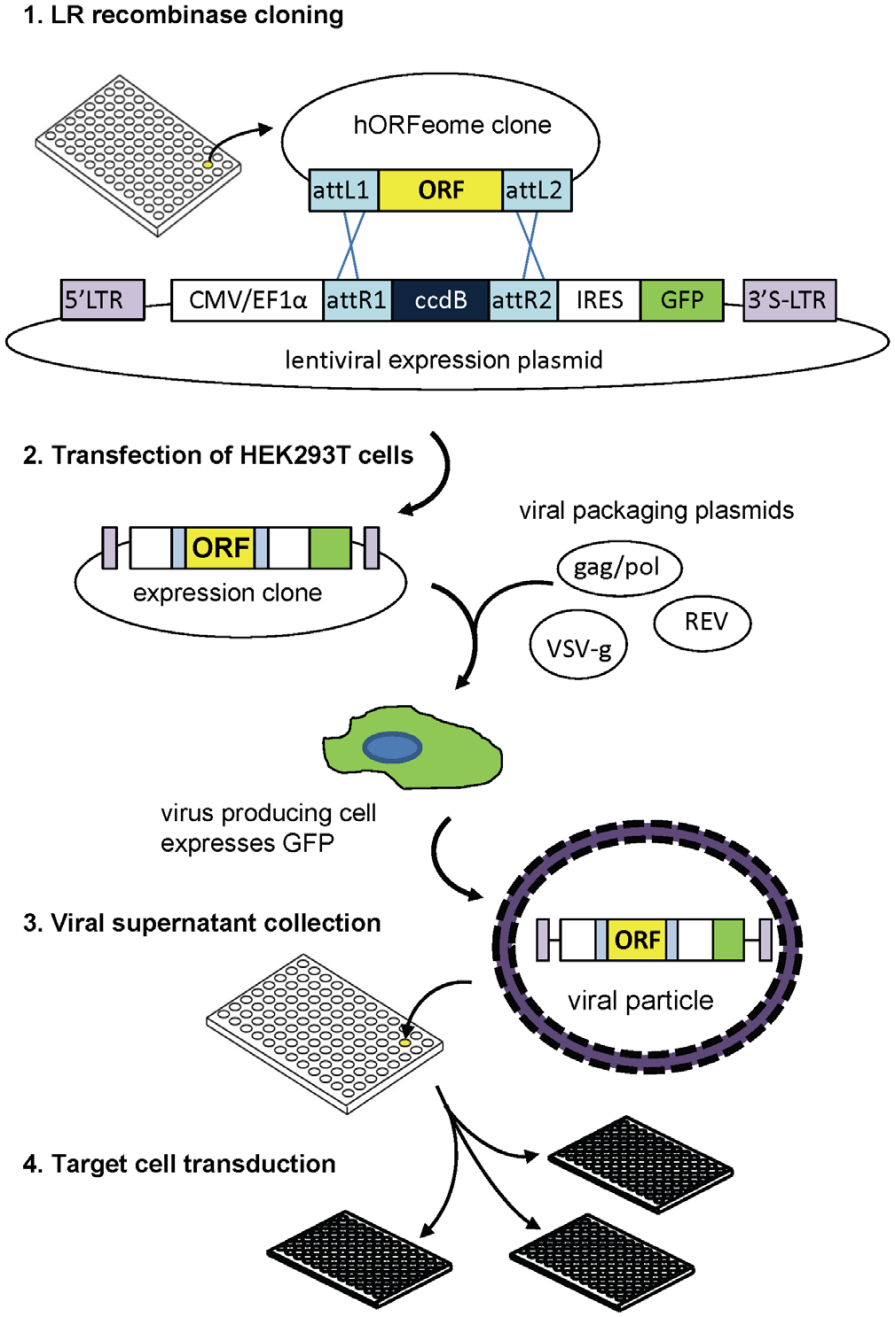
Lentiviral expression library generation. Step 1 shows the structures of the plasmids involved in the Gateway LR recombination reaction between the ORFeome entry vector and the lentiviral expression vector. attL1, attL2, attR1, attR2 are the recombination sites,: ORF - open reading frame; LTR - long terminal repeats (“S” in the 3′ LTR indicates that it harbours a deletion rendering it self-inactivating); CMV/EF1α – promoter; IRES – internal ribosome entry site sequence; GFP – green fluorescent protein, ccdB – gene encoding a bacterial toxin (which is replaced by the ORF and is used to select against non-recombinant plasmids). Steps 2, 3 and 4 are viral packaging in 293T cells, collection of lentivirus-containing supernatant and transduction of target cells, respectively. For details see Materials and Methods.

A diagram of the library construction pipeline is shown in Figure 1. All experiments were performed in 96-well microplate format. The first step in generating the human ORFeome lentiviral expression library was transfer of ORFs from the entry vectors into plv101G destination vector using the Gateway LR recombinase reaction. During this step, the human ORF replaces the ccdB gene downstream of a constitutive promoter - CMV for the initial screening, and CMV or EF1α for the hit-validation experiments. The ORF is inserted upstream of the IRES-controlled hrGFP, so that transgene expression can be monitored by GFP fluorescence. We found that the recombinase reaction on the robotic platform could be performed efficiently in a volume of 5 µl/well, with as little as 1 ng of entry clone DNA. Under these conditions, of 600 clones sampled, only 3% either failed to produce plasmid DNA (12 clones) after transformation, or produced incorrectly-sized products (6 clones) in restriction digest analysis.

In the next step, expression plasmids were combined with viral packaging plasmids and transfected into HEK293T cell line to produce virus-containing supernatant. During optimisation experiments we measured whole-well GFP fluorescence using a plate reader to assess levels of packaging cell transfection. This measurement was found to correlate well with the proportion of GFP positive cells measured by flow cytometry and the high content imager (Figure S1). Whole-well GFP fluorescence of virus-producing cells also served as an estimate of the relative amount of virus produced, as it would have been impractical to calculate titres and multiplicity of infection (MOI) values for thousands of samples prior to screening. In general, observed GFP fluorescence of virus-producing cells and titres obtained from the derived viral supernatant varied on average by less than 15% between replicates of the same clone, but values obtained from different transgenes varied by up to a hundred fold (Figure S2). The greatest source of variation in viral titres was the overexpressed ORF. In the primary screen presented here the percentage of GFP positive cells between ORFs ranged from 0 to 79% (Table S1). It is possible that clones that produced low titres code for proteins detrimental to either the virus-producing HEK293T cells or the test MCF-10A cell line. Nevertheless, the titres achieved allowed us to sample enough transduced cells for statistical analysis of 95% of the clones in at least one round of screening. One virus-producing well produced sufficient supernatant for infection of 6 wells of assay cells, allowing for repeat experiments and/or multiple assays from a single virus production run.

### Overexpression screen

**Figure 2:**
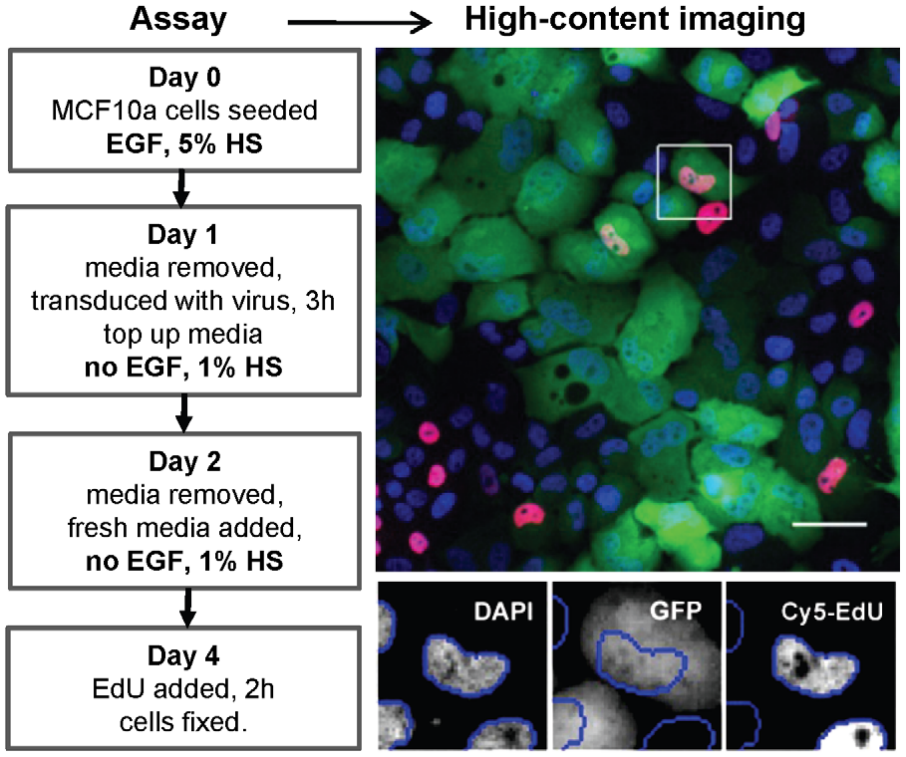
Workflow for the screening of the MCF-10A cells. The left-hand panel shows plate manipulations and cell treatments for each day of the screen. The right-hand panel shows a representative image of a scanned field as a pseudo-colour overlay (top) of individual scanning channels (bottom) shown individually for the boxed area (DAPI – blue, GFP – green, EdU – red, scale bar = 50 µM). Blue lines in the enlargements encircle the object (nucleus) area selected by the scanning algorithm. See Materials and Methods for further details.

The virus generated from the 1990 expression clones was used to assess the ability of the overexpressed ORFs to induce increased proliferation in the MCF-10A cells after EGF removal. In preliminary experiments in the 96-well microplate format we determined the optimal assay conditions that significantly reduced cell proliferation but did not significantly reduce cell viability after exposure to the virus. Under these conditions cells not exposed to virus undergo 2–3 division cycles after EGF withdrawal before depleting the media and arresting in the G1 phase of the cell cycle. As summarized in Figure 2, cells were plated at 2000 cells per well in complete media (containing EGF and 5% v/v horse serum) on Day 0, and transduced on Day 1. Medium without EGF and containing 1% v/v serum was used for subsequent volume top-up and a Day 2 medium change. On Day 4, cells were pulsed for 2 h with the nucleotide analogue EdU which is incorporated into the nuclear DNA of cells in S phase. Cells were then fixed and processed for high-content imaging by staining with DAPI to define nuclei and measure DNA content, and by cross-linking EdU with the fluorescent label Cy5 to mark S phase nuclei. Plates were then scanned on a high-content imager, and individual nuclei scored for DAPI, GFP and Cy5 fluorescence, based on cumulative total (DAPI, Cy5) or average (GFP) nuclear pixel intensity.

### Analysis of screening data and hit selection

**Figure 3:**
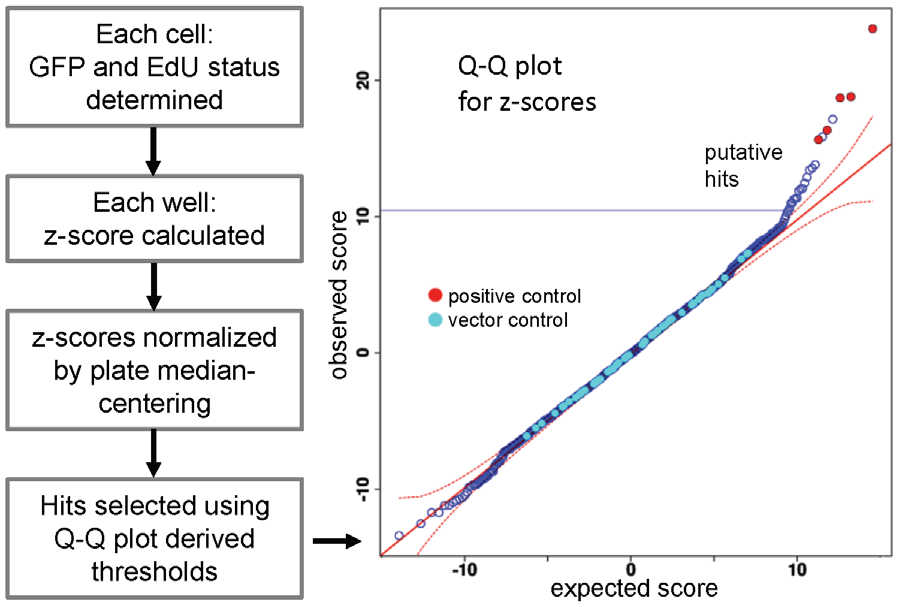
Data analysis steps used to identify hits. Image analysis data was exported from the Cellomics ArrayScan instrument and further analysed using the R programming platform. The first step in analysis is assignment of Edu and GFP status to each cell, (See also Figure 2 and Figure S3). Following further data processing (see Materials and Methods), hits were selected based on Q-Q plots for z-scores. The expected z-score distribution (x axis) is plotted against the observed scores (y axis), and a hit threshold (blue solid line) set where observed values are outside the 95% confidence interval (red dashed line) from the expected y = x diagonal (red solid line). Empty vector negative controls (light blue circles) lie on the diagonal, while the positive control wells, containing cells overexpressing CCNE1 (red circles), are in the predicted hit region.

Image analysis data were further processed using software scripts written in the R programming language (http://cran.r-project.org/), as summarized in Figure 3.The first step in the analysis was to determine the GFP and EdU status (positive or negative) of each cell based on observed fluorescence intensity. To compensate for the artefacts due to uneven background staining and well crowding, this was done using robust regression methods, as detailed in Materials and Methods, and Figure S3.

The second step was to test whether EdU incorporation rates in the transduced (GFP positive) cells were higher than in the untransduced (GFP negative) population in the same well using a z-score based on a binomial distribution. As there was considerable variability between plates, z-scores were then normalized by plate median centering. To obtain z-score thresholds for hit selection, the observed z-score distribution was compared to the predicted normal distribution for a similar mean and standard deviation using Q-Q plots (Figure 3). Thresholds were visually determined by selecting values that lie above the 95% confidence interval from the predicted y = x diagonal. Each plate contained at least 3 wells transduced with empty-vector plv101 which was used as a negative control. As a positive control we used wells transduced with cyclin E1 (CCNE1)-expressing lentivirus, as this gene significantly increased EdU incorporation rates in preliminary experiments. As expected, the negative control plv101 vector control scores are distributed along the y = x diagonal, while the CCNE1 positive control z-scores lie significantly above the line (Figure 3).

When the data were analysed in this way, five out of 144 vector control wells were picked as hits (false positive rate = 3.5%), while one in 15 CCNE1 wells was missed in two rounds of screening (false negative rate = 6.7%). Since this indicated that our false negative rate exceeded the false positive rate, and since this was a proof-of-principle pilot screen, we chose to include in further analysis 128 clones, 106 of which behaved as hits (based on Q-Q plots) in at least one round of screening and 40 of those were hits in both rounds (Table S2). A further 22 non hit clones were selected to further assess the specificity of the screen and analysis.

### Confirmation and characteristics of hits from the primary screen

Since the ORFeome collection clones were originally derived from PCR products and thus potentially contained multiple DNA species, we remade virus from three single colony isolates for each of the 128 selected genes and passed them through another round of screening. In this secondary/validation screen, z-scores were normalized against empty vector controls. The inserts of the high-scoring clones were sequenced and confirmed to match the sequence of the corresponding MGC clone indicated in Table S1. The 47 genes for which at least two of the three colonies were verified as hits in the EdU assay are listed in Table 1.

**Table 1:** Identity and characteristics of confirmed EdU incorporation hits: function criteria, cell-cycle expression pattern and activity in related functional screens.

Symbol	Description	Function criteria*	Cell cycle phase†	Functional screens§
APEX1	APEX nuclease (multifunctional DNA repair enzyme) 1	ML		1−,2−
ATOX1	ATX1 antioxidant protein 1 homolog	U		6
BUB3	budding uninhibited by benzimidazoles 3 homolog (yeast)	C	M^a^,G2^b^	4b
C2orf83	chromosome 2 open reading frame 83	U		
CAPN3	calpain 3, (p94)	ML		
CDC20	cell division cycle 20 homolog	C	M^a^, G2^c^	4a,4b
CDK2	cyclin-dependent kinase 2	MR, P		1−,5
CDK9	cyclin-dependent kinase 9	C, P	G2^a^	1+,2+,3+
CELA2B	chymotrypsin-like elastase family, member 2B	U		
CLDN1	claudin 1	ML		
CLK2	CDC-like kinase 2	P		1+
CRK	v-crk sarcoma virus CT10 oncogene homolog	C	M/G1^b^	1+,2+
CYTH2	cytohesin 2	C	G2^ab^	1−
DCAF7	DDB1 and CUL4 associated factor 7	C	Ma,G2^b^	2+,3
DPYSL3	dihydropyrimidinase-like 3	C	G2^a^	2−
EXOC8	exocyst complex component 8	C	S^a^	
GAPDH	glyceraldehyde-3-phosphate dehydrogenase	C	G2^a^	
GNA15	guanine nucleotide binding protein (G protein), alpha 15 (Gq class)	C	M^a^	3
GNB1	guanine nucleotide binding protein (G protein), beta polypeptide 1	C	S^a^,M/G1^b^	2+,3
H2BFWT	H2B histone family, member W, testis-specific	U		
HNRNPH2	heterogeneous nuclear ribonucleoprotein H2 (H′)	U		
HOXB5	homeobox B5	U		
ILKAP	integrin-linked kinase-associated serine/threonine phosphatase 2C	P		
IRAK3	interleukin-1 receptor-associated kinase 3	U		
KRT19	keratin 19	C	G2^a^	1−,2−
KRT40	keratin 40	U		
MAK	male germ cell-associated kinase	P		
NAB2	NGFI-A binding protein 2 (EGR1 binding protein 2)	C	S^a^	
NDUFS3	NADH dehydrogenase Fe-S protein 3, 30 kDa	P		
NEK6	NIMA (never in mitosis gene a)-related kinase 6	P		2+
PACSIN1	protein kinase C and casein kinase substrate in neurons 1	U		
PDK2	pyruvate dehydrogenase kinase, isozyme 2	P		4a
PPM1G	protein phosphatase, Mg2+/Mn2+ dependent, 1G	MR, P		5
PSTPIP2	proline-serine-threonine phosphatase interacting protein 2	ML,		5, 6
RBP1	retinol binding protein 1, cellular	U		
RGS20	regulator of G-protein signaling 20	C	M^a^	
RIT1	Ras-like without CAAX 1	U		
RPS6KA4	ribosomal protein S6 kinase, 90 kDa, polypeptide 4	MR, P		5
SETD2	SET domain containing 2	P		
SH3GL2	SH3-domain GRB2-like 2	C	S^b^	
SOD1	superoxide dismutase 1, soluble	ML		4a,4b
SPOP	speckle-type POZ protein	C	lateG1^a^	2+,3,4a
SYT5	synaptotagmin V	MR		6, 5
TK1	thymidine kinase 1, soluble	MR		5
TMEM55A	transmembrane protein 55A	C	S^a^	
UBE2S	ubiquitin-conjugating enzyme E2S	C	M^a^,M/G1^b^,G2^c^	
WWP2	WW domain containing E3 ubiquitin protein ligase 2	C	S^a^	2+,4a
ZWINT	ZW10 interactor	C	S^b^	4b

*Function criteria used for inclusion in the lentiviral ORF library, based on published microarray expression pattern or functional screening result: C- cell cycle phase-specific expression; ML-Myeloid cell proliferation [26]; P- phosphoregulation [27], [28]; MR- mitotic regulation [24]; U- unspecified i.e. randomly picked clones.

† Cell cycle phase-specific expression determined by microarray analysis in: **a**, primary human foreskin fibroblasts [22] or HeLa cells **b**, [25] and **c**, [23].

§ Positive (+), negative (−) or neutral (*) effect on cell proliferation detected in either cDNA overexpression screens in: **1**, SMMC7721-human hepatoma cells [11]; **2**, NIH 3T3 mouse fibroblasts [11]; **3**, U2OS-human osteosarcoma cells [9]; or in RNAi-mediated knock-down screens for effects on mitosis detected in **4a-** HeLa [29], **4b-** Hela [32], **5-** HT29-human colon carcinoma [24], and **6-** U2OS cells [31].

These validated hits include 10 (1.5%) of the randomly selected genes (Functional criterion U, Table 1) and 37 (2.8%) of the genes preselected for screening based on their putative function (C-cell cycle, P-phosphoregluation, ML- myeloid cell proliferation, MR- mitotic regulation). Of the 19 hits that were included based on demonstrated periodic cell cycle expression pattern, only three were specifically associated with G1 phase. Comparison to published functional screens specifically addressing cell cycle and proliferation in human cells revealed that knockdown of 12 of our hit genes produced a cell cycle defect in other systems [24], [29], [30], [31], [32] while the overexpression of CDK9, CRK, and CLK2 also increased proliferation in human hepatoma cell line SMMC7721 [11]. CDK 9 overexpression also increased proliferation of human osteosarcoma cell line U20S2 [9], while CRK, CDK9, DCAF7, NEK6, GNB1, SPOP, and WW2 also increased proliferation in mouse fibroblasts [11]. In contrast, overexpression of APEX1, CYTH2, CDK2, DPYSL3 and KRT19 has been reported to decrease proliferation in other cell lines (Table 1) [9], [11]. However, it should be noted that the CDK2 cDNA used by Harada *et al* [9] encoded an extensively truncated protein and thus may not be comparable.

When we compared the list of hits to the background of screened genes using the Functional Annotation Clustering tool on the DAVID Bioinformatic Resources web site [33], no significant enrichment for any functional cluster was found. This is not surprising, since our background set was biased towards genes involved in cell cycle, phosphoregulation and myeloid cell proliferation. Additional broad functional categories identified by DAVID tool containing multiple hits included nucleotide and nucleic acid metabolism (APEX1, CDK2, CDK9, CRK, DPYSL3, HNRNPH2, HOXB5, IRAK3, NAB2, RPS6KA4, SETD2, SOD1, SPOP, TK1), cell-surface receptor signalling (GNA15, GNB1, IRAK, RGS20), cell proliferation (BUB3, CDK2, CDK9, GNB1, IGSF4, NAB2), membrane organisation (CYTH2, PACSIN1, SH3GL2, SOD1), actin cytoskeleton organisation (CRK, CYTH2, KRT19, SPOP), and protein catabolic process (BUB3, CAPN3, CDC20, IRAK3, SPOP, UBE2S, WWP2). Two of the hit genes, C2orf83 and CELA2B, have only been electronically annotated and no functional studies have so far been reported. CAPN3 was represented by two independent clones, representing different splice variants, adding confidence to its hit status.

### Proliferative activity of validated hits

**Figure 4:**
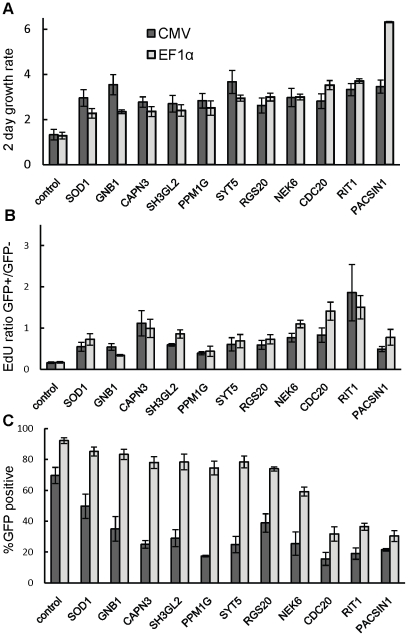
Summary measurements for hits showing increased total cell proliferation. CMV, EF1α indicate values for ORFs expressed under control of the CMV and EF1α promoters, respectively. A – ratio of total objects counted on day 4 over day 2 of the assay (Figure 2); B – ratio of the proportion of EdU positive cells in GFP positive over that in GFP negative objects on day 4; C-percentage of GFP positive objects on day4. All bars represent a mean of three wells, error bars are standard deviation. Genes represented here were significantly different (p≤0.05) in A and B from the control (in this case vectors encoding a truncated peptide originating from CPNE3, which had no effect on proliferation in primary and validation screens). Data for *all* genes tested is in Table S3.

From the multiple rounds of screening we identified 47 genes that satisfy criteria for increased nucleotide incorporation rate, suggesting that they may be increasing cell proliferation after EGF removal. We took advantage of the flexibility of our platform to further analyse the effect of hit ORFs on cell proliferation rate and cell cycle profile. These hit ORFs were also subcloned into alternative lentiviral vector plv411G, which is identical to plv101G except that it utilises the EF1α promoter in place of the CMV promoter. The EF1α promoter presented advantages over the CMV promoter when we generated stable cell lines overexpressing transgenes. In the MCF-10A cell line background we detected silencing of the CMV promoter, while the EF1α promoter maintained stable expression through multiple cell passages and at least one freeze/thaw cycle (data not shown). With these two sets of expression clones we performed a time course experiment similar to the assay described in Figure 2, except that triplicate plates were EdU labelled and harvested on days 2 and 4 (i.e. 1 and 3 days respectively after transduction and EGF withdrawal). This allowed us to measure the effect of transgene overexpression on the increase in cell number, as well as to observe the changes in cell cycle induced by transduction and EGF withdrawal. As a negative control we used a clone expressing a 24 amino acid truncated version of CPNE3 which produced neutral z-scores in screens described here, as well as in previous preliminary experiments. The results for all 47 genes are presented in Tables S3 and S4, while the data for the 11 genes that significantly (P≤0.05) increased both cell number and EdU incorporation rate compared to control (see below) are shown in Figure 4.

In general, the CMV promoter drove stronger GFP expression, detected as more intense fluorescence which was detectable earlier compared to EF1α driven expression. On day 2 (26 hr post- transduction), the median number of GFP positive cells per well across the 3 test plates was 850 with the CMV promoter compared to only 220 with the EF1α promoter. At this time 26% of EF1α driven transgenes produced less than 50 GFP positive cells per well, making them unsuitable for statistical analysis. In contrast, on Day 4, wells transduced with EF1α driven transgenes had more GFP positive cells, with a median of 5214 compared to 2771 median GFP positive cells per well produced by CMV driven transgenes. This corresponded to 74% and 35% respectively of total cells counted per well (Figure 4C, Table S3).

Since proliferation rates of transduced cells in this experiment could not be accurately determined given the changes in GFP expression, we calculated the increase in total cell numbers in transduced wells between days 2 and 4 (Table S3). Eleven genes induced significantly (P≤0.05) larger increases in cell number compared to the control, regardless of the promoter used (Figure 4A), indicating that they can stimulate cell proliferation. Of the others, ATOX1, APEX1, CDK9, H2BF2 induced smaller increases in cell number compared to the control with the CMV promoter, while MAK and CADM1 failed to produce at least 50 GFP positive cells by Day 4. All others showed growth rates that were equal or slightly higher but not statistically significantly different from those of the control and three other non-hits tested (Table S3). Cells in untransduced wells, or cells in wells exposed to the mock supernatant (containing empty viral particles only) exhibited higher growth rates than in any ORF-expressing wells, indicating that ectopic protein expression or transduction *per se* had an anti-proliferative effect (data not shown). The ratio of EdU incorporation rates for transduced over untransduced cells was significantly higher for the hits analysed in this experiment compared to the control (Figure 4B, Table S3), reconfirming that the hits behaved as observed in the previous rounds of screening and making it unlikely that the observed increase in proliferation was caused by the untransduced cells. This is further supported by the fact that of the 11 proliferation hits, eight had transduction rates of 60% or more with the plv411 vector.

**Figure 5:**
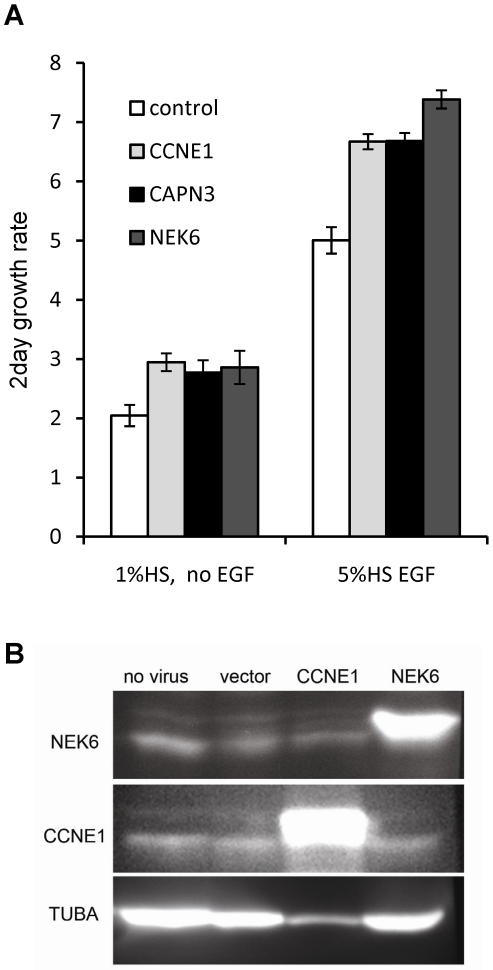
Effect of transgene overexpression on 2-day growth rate of FACS-purified cell populations. MCF-10A cells were transduced with control (truncated CPNE3), CCNE1, CAPN3 or NEK6 vectors and sorted by flow cytometry for GFP expression (minimum 98% GFP positive cells). A- Cells were seeded in complete medium (5%HS and EGF), and the next day media were replaced with either reduced (1% HS no EGF) or complete medium. Cells were counted 1 and 3 days later and ratios calculated. Bars represent mean of 4 wells and error bars show the standard deviation. B – Western blots of whole cell protein extracts obtained from sorted cell lines after one freeze-thaw cycle and 8 subsequent passages. Targeted antigen is indicated on the left of the image while the introduced ORF is indicated on top of each lane. Except for the no virus control, cells used were confirmed to be >98% GFP positive prior to harvesting.

We tested the effect of the overexpression of two high confidence hits, CAPN3 and NEK6 in cell lines that have been sorted by FACS to generate a pure population of transduced cells. Cells were sorted 8 days after transduction and seeded in complete (5% HS, EGF) medium. After 48 h the medium was removed and replaced with either complete medium or medium with no EGF and 1% HS. Both CAPN3 and NEK6 overexpression induced a significant (p<0.05) increase in growth rate compared to control cells (Figure 5A). The increase was comparable to that conferred by the CCNE1 positive control, and was observed in both complete and growth-factor reduced media, indicating that the pro-proliferative effect of these transgenes is not restricted to overcoming G1 arrest caused by EGF withdrawal. However, by four weeks following transduction, this increase could no longer be detected (data not shown). The overexpressing cell lines remained more than 98% GFP positive and continued to express the introduced ORF at above normal levels (Figure 5B) through at least one freeze-thaw cycle and up to 8 subsequent passages, indicating that the loss of effect was not due to a reduction in protein expression.

**Figure 6:**
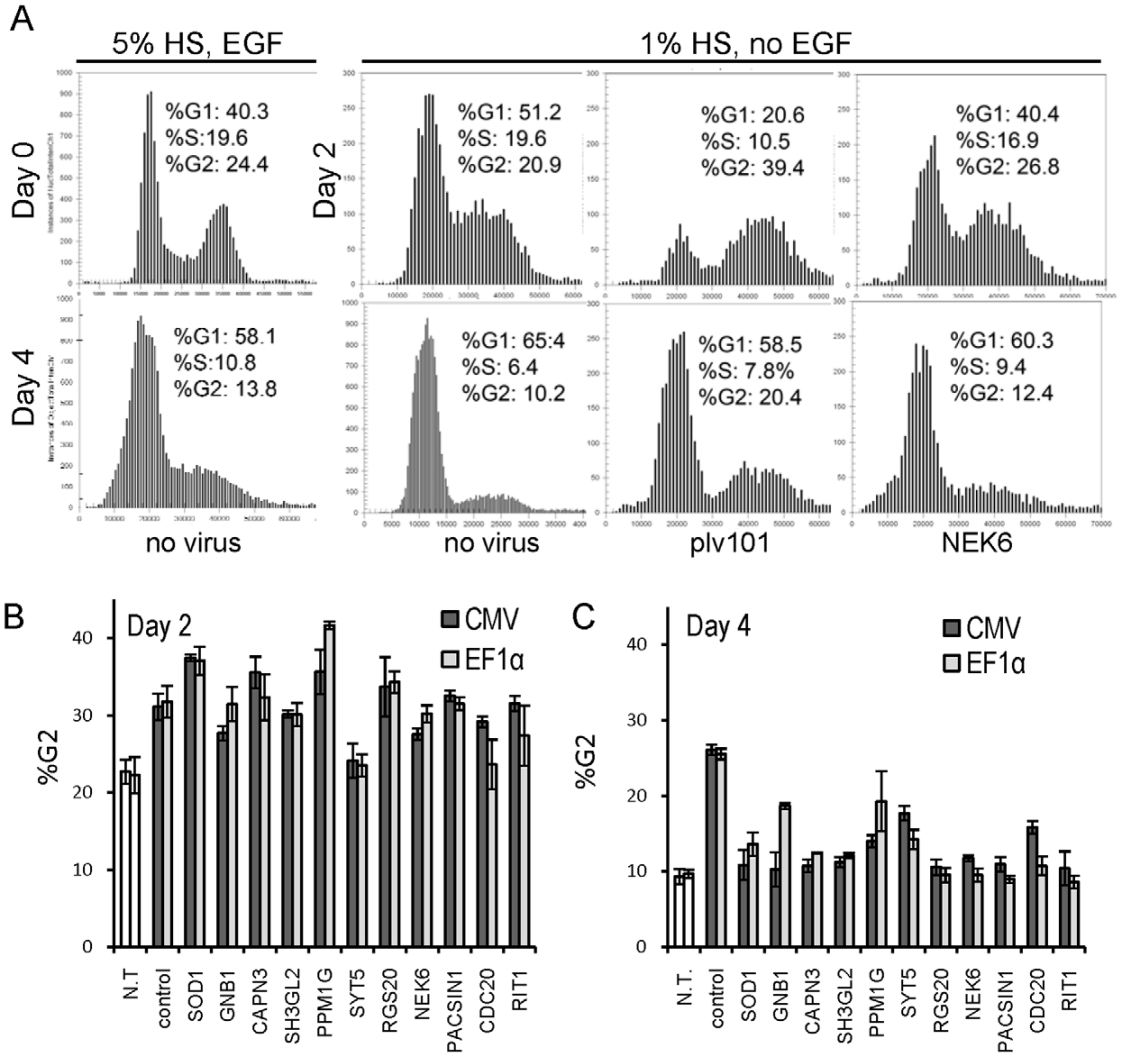
Cell cycle analysis of proliferation-inducing hits. A- DAPI intensity (x-axis) histograms (y-axis = number of objects) obtained by CellCycle v3 application in the Cellomics ArrayScan Software, representing typical profiles observed on Day 0, Day 2 and Day 4 of the assay (Figure 2), depending on growth conditions and viral transduction. Profiles and data shown are derived from a representative single well from each of the categories: not transduced (no virus), transduced with empty plv101 vector, or with the vector expressing NEK6, analysed using cells grown in complete or restrictive medium as indicated. The proportion of cells in each cell cycle phase is indicated (based on DNA content: 2N (%G1), between 2N and 4N (%S), and 4N (%G2)). B, C – Graphs representing the proportion of cells in G2 on day 2 (B) and day 4 (C) for proliferation-inducing hits (Bars = mean of 3 wells; error bars = standard deviation). CMV, EF1α - values for clones expressed under control of the CMV and EF1α promoters respectively. N.T-not transduced, control – truncated CPNE3. Values for all analysed genes are in Table S4, for all transduced wells, values represent GFP positive cell population only.

Cells in our screen were exposed to two antiproliferative conditions: EGF withdrawal which causes accumulation of cells in G1 phase of the cell cycle [21] and lentiviral infection which has been shown to promote G2 phase accumulation [34], [35]. To determine which of these antiproliferative conditions the hit transgenes were overcoming, we examined cell cycle progression in transduced cells by DNA content analysis. Using the DAPI intensity histograms obtained by high-content imaging, nuclei were assigned cell cycle phases based on their deduced DNA content (G1 = 2N, G2 = 4N, and S = (2N–4N)), by modelling on the histograms derived from the reference untransduced wells (Figure 6A). Three hours after plating, cells that had not been exposed to viral supernatants and were grown in complete media had a mean G1/G2 ratio of 1.6±0.13 (n = 12) (Figure 6A). On day 2 (Figure 2) of the assay, after a medium change to 1% serum without EGF, cells in untransduced wells started accumulating in G1 (G1/G2 = 2.1–2.4). In contrast, most wells exposed to viral supernatant had an increased proportion of cells in G2 phase compared to cells in untransduced wells (Figure 6A, B; Table S4). Fewer cells were observed in the transduced wells at this time, indicating that the G2 accumulation was due to G2 block rather than faster progression through G1/S. This effect was observed with both CMV and EF1α promoters, and was detected in both GFP positive and negative cells in transduced wells, as well as in cells exposed to mock supernatant (from cells transfected only with the packaging vectors), suggesting that it may be caused by both empty viral particles as well as the particles containing the ORF expressing RNA. Irrespective of the promoter used, the G2 phase accumulation was less pronounced in cells overexpressing hit genes CADM1, CDC20, CDK2, CELA2B, CLK2, KRT19, NEK6, PACSIN, or RIT1 compared to control and other hits (Figure 6, Table S4). On Day 4 of the assay (Figure 6A, and C), untransduced cells growing in EGF-free media were arresting in G1 phase, evident from the reduced proportion of cells in S phase and an increased proportion in G1. This EGF withdrawal-induced G1 arrest was more pronounced and happened at lower cell densities compared to the G1 accumulation observed in untransduced cells in complete media that almost reached confluence (Figure 6A). In contrast, cells transduced with the truncated CPNE3 control still had a significantly higher proportion of cells in G2 phase compared to those in untransduced wells, although not as high as on day 2. Cells transduced with hit genes had G2 proportion values between those of the transduced control and untransduced wells in EGF withdrawal (Figure 6A, C, Table S4), indicat ingthat at least in some cases transgene overexpression compensated for the transduction induced G2 phase arrest observed on Day2. For most hit genes, the proportion of cells in G1 phase was lower compared to the untransduced controls subjected to EGF withdrawal (Table S4), indicating that these genes may also compensate for growth factor removal.

## Discussion

We have described the development and successful application of a platform for gain of function screening of large numbers of human protein-coding genes. The platform brings together optimised high-throughput approaches for recombinational cloning, lentiviral production, mammalian cell transduction and high-content imaging, and thereby expands the scope of functional genomics questions that can be experimentally addressed.

Here we have presented construction and testing of a lentiviral expression library of 1990 human ORFs in arrayed single gene per well format. This library has since been expanded to encompass 17,500 clones, which will be described elsewhere. Several features of these libraries maximise utility and efficacy. First, since lentiviral vectors pseudotyped with the VSV-G envelope protein have been successfully used to introduce genes into a broad variety of mammalian cell types [36], [37], [38] in applications including arrayed high-throughput shRNA screens [3], [24], [39], our library expands the scope of gain-of-function screening. Second, the optimised high-throughput Gateway cloning enabled robotics-assisted generation of expression clones and will facilitate clone transfer to vectors that may contain different features. For example, the Gateway-compatible lentiviral vector series described by Campeau *et al* [16] allows addition of different promoters, selectable markers and tracking tags to the overexpression clone. We demonstrated the utility and feasibility of this feature by recloning hit ORFs into a vector with an alternative promoter. Third, the lack of non-coding sequences in our library enhances ORF expression since it eliminates interference by the gene-specific regulatory sequences that may be contained in untranslated regions of some cDNA clones. Fourth, a primary screening vector in which ORF expression, driven by the CMV promoter, is from the same transcript as an IRES-driven GFP. In this construct, neither ORF nor GFP expression is affected by frame shifts that are sometimes caused by slippage in *att* recombination sites during generation of entry clones [40]. Monitoring transgene expression by GFP fluorescence on the high-content imaging platform, rather than introducing a selectable marker is advantageous since it allows for comparison of transduced and non-transduced cells in the same well. It also avoids potential toxic effects of the selection agent as well as large variations in the total cell number per well. We have demonstrated that GFP fluorescence of transduced cells correlates with significantly increased target protein expression by both quantitative immunocytochemistry at a cell-based level, as well as in Western blots of proteins from lysates of cells sorted for GFP expression. Finally, the use of a high-content imager, which can rapidly scan and measure thousands of cells per well, allowed us to obtain meaningful data from wells where viral transduction rates were less than 1 percent, obviating the need to concentrate the virus. This feature will be particularly useful in cell lines which are more difficult to transduce. In our pilot screen less than 5% of the clones failed to produce sufficient titres for analysis. Similar results were obtained in an unrelated screen using MCF-7 cells (unpublished observations). Moreover, use of the test gene set shown in Figure S2 on HaCaT and MDA-MB-231 cells again gave a similar range of transduction frequencies (data not shown). We have identified 47 genes whose overexpression can increase the rate of nucleotide incorporation into DNA in MCF-10A cells in the absence of EGF. In addition to genes associated with the cell cycle, phosphoregulation and myeloid cell proliferation which had been enriched in the screening set, the hits included genes with functions in nucleic acid metabolism, signal transduction, cytoskeletal functions and membrane and protein processing. This broad range of gene functions that can affect rates of nucleic acid synthesis, and potentially proliferation rates, is indicative of the complexity of these cellular processes. Comparison of our results with two published functional screens targeting mammalian cell proliferation [9], [11] has revealed that some genes may be universally pro-proliferative (eg. CDK9), while others may have different (eg DCAF7, SPOP) and even opposing (eg. APEX1, KRT19) effects depending on the system studied. Similar diversity has been observed between loss of function screens targeting the cell cycle in different cell types, where the reported hit overlap was between 6 and 36% [29], [32]. Functional studies of individual genes indicate that this is not a reflection of poor reproducibility of high-throughput screening, but a consequence of functional promiscuity of some genes. Among our hits this is exemplified by NEK6, RIT1, PACSIN and CAPN3, which may have multiple functions depending on expression context [41], [42], [43], [44], [45], [46]. In contrast, BUB3, ZWINT and UBE2S have highly conserved roles in the cell cycle across many cell systems studied [47], [48], [49], [50], [51].

Cell proliferation rate is a quantitative trait dependent on many extrinsic as well as intrinsic factors. In the high-throughput screening context, forced overexpression of any gene might be expected to affect proliferation rate to some extent, generating a basal level of variability. This variability is further compounded by technical factors influencing the well microenvironment, such as minor differences in volume of liquid transfer or position on the plate, so that any gene identified as a hit must have sufficient pro-proliferative power to be detected above this level of background noise. We compensated for this by analysing the screen using z-scores where the control value is derived from non-transduced, i.e. GFP negative, cells in the same well. The caveat of this approach is that secreted proteins or proteins that can increase proliferation in neighbouring cells through paracrine pathways may result in false negatives. False negatives may also result from wells with high transduction rates in which a certain proportion of cells identified as GFP negative, will be expressing the ORF due to the generally lower expression of proteins following an IRES [52], [53], [54], and delays in production of sufficient GFP for detectable levels of fluorescence.

Of the 47 hits that increased the EdU incorporation rate 2 days after transduction and EGF withdrawal, 11 also caused a detectable increase in cell proliferation rate irrespective of the promoter used to drive transgene expression. It is possible that the genes that affected nucleotide incorporation but not cell proliferation rate act on pathways that are specific to nucleotide metabolism and/or DNA repair (eg. TK1, APEX1). Alternatively, the effect of particular transgenes on cell proliferation may have been temporary and/or too small to affect the cell numbers over the course of the screen, but sufficient to cause a detectable increase in EdU incorporation rate during the 2 hours of the assay. CAPN3 and NEK6 overexpression also caused increased proliferation rate in stably overexpressing cell lines isolated by FACS. However, this increase was sustained for only a few passages after sorting despite continued transgene overexpression, suggesting that MCF-10A cells are capable of restoring proliferation control.

Our pilot screen was performed after EGF removal and under reduced serum concentration in order to target genes that can overcome G1 arrest. Because we chose assay conditions that allowed 2–3 rounds of cell division to occur before the G1 phase arrest, it is not altogether surprising that we identified as hits both genes that have known G1/S phase promoting activity such as RIT1 [46], CRK [55], and CDK2 [56], as well as genes that have documented roles in G2/M phase progression such as CDC20 [49], NEK6 [45], [57], [58], BUB3 [51], ZWINT [50], and UBE2S [47], [48], [49]. Cell cycle analysis indicated that this is in part due to the fact that the hit transgenes may be overcoming G2 phase block caused by exposure to viral supernatant, instead of, or in addition to the G1 phase block caused by growth factor withdrawal. Although our analysis suggests that this is true for at least CADM1, CDC20, CDK2, CELA2B, CLK2, KRT19, and NEK6, further experiments are required to confirm the mechanism of transgene action. Future whole genome screens could be designed with more stringent conditions to target a specific cell cycle phase or molecular pathway.

In conclusion, we have described protocols for the construction and screening of a lentiviral ORF overexpression library, which we have successfully tested in a screen for pro-proliferative genes. The identification of hits that include genes previously known to have this activity, as well as novel genes, demonstrates the utility and relevance of our lentiviral overexpression screening platform and provides directions for future more detailed functional analysis of identified genes. This gain-of-function screening platform complements the siRNA and shRNA depletion screens currently available, and provides a powerful new approach for high-throughput functional genomics.

## Materials and Methods

Unless otherwise noted, all reagents were obtained from Sigma-Aldrich (Sydney, Australia).

### Plasmids, bacterial strains and cells

Bacterial cultures of entry clones in pDONOR223 or pENTR201 vectors were picked respectively from the Human ORFeome collection version 1.1 and 5.1 or from the Human Orfeome collaboration OCAA collection (Open Biosystems), and arrayed as indicated in Table S1. Gateway-cloning-compatible lentiviral expression plasmids plv101G and plv411G, and the lentiviral packaging plasmids [59] pRSV-Rev, pCMVdelta8.2 and pVSV-G were obtained from S. Barry [60].

Entry clones were maintained in *E.coli* strain as supplied, in LB/TB (1∶1) medium supplemented with spectinomycin (50 µgml^−1^). Expression clones were transformed into α-Select Gold chemically competent cells (Bioline), and maintained in 100 µgml^−1^ ampicillin in LB/TB. Cells were cultured in 96-deep-well Costar 3960 plates(Corning) in Higro™ multiplate incubator-shaker (Digilab), and stored in 96-well Costar 3896 plates (Corning) in media containing 10% glycerol at −70 C.

HEK293T (Broad Institute, Cambridge MA) cells were maintained under standard tissue culture conditions in DMEM supplemented with 10% (v/v) heat-inactivated fetal calf serum (Hyclone), 0.85 mM HEPES, 2 mM L-glutamate, 1 mM sodium pyruvate, 1× non essential amino acids (GIBCO).

MCF-10A cells (ATCC) were maintained in DMEM/F12 (1∶1; Invitrogen) supplemented with 5% (v/v) heat-inactivated horse serum (Invitrogen), 10 µg/ml insulin, 20 ng/ml EGF, 0.5 µg/ml hydrocortisone (Bayer), 100 ng/ml Cholera toxin, and penicillin/streptomycin antibiotic (Invitrogen).

### Robotic platform and plate handling

Multistep micro-plate protocols were performed with SciClone ALH3000 liquid handling workstations (Caliper Life Sciences; Hopkinton, MA, USA). Separate workstations were set up for DNA and mammalian tissue culture experiments. Plate-washing steps were performed with an ELx405 plate washer (BioTek Instruments, Winooski VT, USA). Single 96-well liquid dispensing steps, including cell seeding were performed with a Matrix Wellmate (Thermo Scientific).

### Expression clone generation

Entry clone plasmid DNA was isolated using Perfectprep plasmid 96 VAC kit (5 Prime) according to manufacturer's instructions, either manually using vacuum manifold (Eppendorf) or on the SciClone ALH3000 robotic platform. Entry clones were transferred into the expression vector using Gateway LR clonase II enzyme mix (Invitrogen). The LR reactions were performed overnight at room temperature in 96-well plates in a volume of 5 µl per well, containing 1–100 ng of entry clone DNA, 100 ng expression plasmid DNA and 0.6 µl enzyme mix in 3 mM Tris-HCL pH 8. Reactions were stopped by addition of 3 µl of Protease K (Invitrogen) diluted in water 1∶1 (v/v). After 20 min at 37 C, protease was heat inactivated at 90 C for 5 min. 2 µl of this was transformed into 200 µl of α-Select Gold chemically competent cells (Bioline) using a 45 s heat shock at 42 C. DNA was extracted from the expression clones cultured in 2 ml LB/TB, as described above. DNA concentrations in stock plates were determined using a Powerwave microplate spectrophotometer (BioTek) and normalised to 20 ng µl^−1^ during a robotic transfer into a fresh set of plates that served as source plates for transfection.

### Virus production in HEK293T cells

Expression clone DNA (300 ng) was mixed with packaging plasmids pRSV-Rev (150 ng), pCMV delta R8.2 (180 ng) and pVSV-G (120 ng) in 19 µl per well in Costar 3896 plates. Lipofectamine™ 2000 transfection reagent (Invitrogen) was incubated with OPTIMEM (Gibco) (1∶ 31, v/v) for 20–40 min at RT, prior to adding to the DNA mix at 31 µl per well. HEK293T cells were plated in 96-well SpectraPlates TC (Perkin Elmer) coated with 0.1% gelatin, at 60000 cells in 200 µl per well. Cells were allowed to settle for at least 2 h, before 50 µl of the DNA-Lipofectamine™ mix was added per well. The next day, 150 µl of medium was aspirated from each well and replaced with medium containing 1 mM Sodium Butyrate. After 48 h, virus (180 µl from each well) was harvested into Costar 3896 plates and stored at −70 C until use. Cells remaining in transfection plates were washed in PBS, and fixed with 10% formalin. To confirm virus production, plates were scanned for total well GFP fluorescence using Fluostar OPTIMA (BMG Labtech) micro-plate reader.

### Transduction and screening of MCF-10A cells

MCF-10A cells were seeded in complete media in black, clear-bottom 96-well Viewplates (PerkinElmer), at 2000 cells in 130 µl per well. The next day medium was aspirated such that 20 µl per well remained. Viral plates were prepared by combining 20 µl of viral supernatant with 9 µl of Polybrene (40 µgml^−1^ in complete medium) in each well; this mixture was then added to each well of MCF-10A cells. Plates were incubated for 2–4 h before 150 µl of low serum media (lacking EGF and containing only 1% serum) was added to each well. The next day 150 µl of media was aspirated from each well and replaced with fresh low serum media. After a 48 hr incubation, cells were pulse labeled with 10 µM EdU (Berry and Associates; Dexter, MI, USA) for 2 h and fixed in 3.7% formaldehyde in PBS. EdU was subsequently cross-linked to Cy5-azide and nuclei were stained with 400 nM DAPI as detailed by Ranall *et al* [21]. Images were acquired with a Cellomics ArrayScan V^TI^ (Thermo Scientific) high-content imager, using a 10× objective and an XF93 filter set.

### Protein blots

Harvested cells were collected by centrifugation, resuspended in SDS-loading buffer, separated by polyacrylamide gel electrophoresis on a 10% gel, and transferred to Hybond-P membrane (Amersham) by semi-dry transfer using BIO-RAD Mini-Protean and Trans-Blot systems according to manufacturers recommendations. Blots were probed with primary gene specific antibodies: mouse monoclonal anti-CCNE1(HE12) (Santa Cruz)1∶500, mouse monoclonal anti alpha tubulin 1∶ 500 (Sigma), or rabbit monoclonal anti-NEK6 (Epitomics)1∶10000; then secondary horse radish peroxidise-conjugated anti mouse (1∶2000) or anti rabbit (1∶2000) sera (Invitrogen). Signal was detected using Western Lighting-ECL reagent (Perkin-Elmer).

### High-content image analysis

Images were analysed using the TargetActivation.v3 application in the Cellomics ArrayScan software. Objects were selected and nuclear area and shape measured in the DAPI channel. Total and average pixel intensity was measured in DAPI, GFP and Cy5 channels and population statistics collected for objects in each well. Data was then exported using the Cellomics Explorer software and further analysed using the R software environment which allowed data manipulation without image rescan.

The first step in the analysis was to determine the GFP and EdU status of each cell. Analysis of EdU and GFP fluorescence intensity revealed that setting simple thresholds introduced false positive and false negative calls caused by variable background staining and cell crowding. These artefacts were eliminated by setting regression-based thresholds as follows:

EdU status: To determine EdU status, we used scatter-plots of total nuclear DAPI vs total nuclear Cy5 fluorescence intensity. As shown in Figure S3A, the variable background staining produced during Cy5-labelling of EdU, resulted in a rising baseline making setting single threshold invalid. To compensate, we performed robust regression using the lmrob function within the “robust regression” library of R (http://cran.r-project.org/). This method fits a linear approximation to the background of Cy5-EdU negative nuclei and identifies outliers, the Cy5-EdU positive cells. In cases where more than 50% of cells are EdU positive, the background of EdU negative cells is fit iteratively using successive rounds of robust regression and excluding the outliers until the regression curve converges with the non proliferating background.

GFP status: In densely populated fields with a high proportion of GFP positive cells, we observed nuclei of non-transduced cells whose edges overlapped with the cytoplasm of GFP positive cells, resulting in above threshold fluorescence in some nuclei of cells not expressing GFP. The false positive GFP cells were identified in plots of log_2_(variance GFP intensity ) vs log_2_(mean GFP intensity) as a distinct cloud of above-threshold GFP signal but with high fluorescence variance (See Figure S3B). These false GFP-positive cells were excluded using a quadratic fit of GFP cells above the background threshold (based on average nuclear GFP pixel intensity) again using the lmrob function in R. EdU and GFP regression analysis plots were automatically generated for all wells in the screen, and visually inspected for putative hit wells to verify that the methods performed as expected. Failures were observed at a frequency of less than 0.5%, and usually generated false positives in wells with small numbers of cells; these were eliminated from the hit list.

### Identification of hits

EdU incorporation rates in the transduced population were calculated using a z-score based on a binomial distribution. In this case the z-score tests whether the proportion of EdU positive cells is different in the transduced (GFP positive) population to that in the untransduced (GFP negative) population for each well:
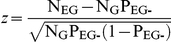
where P_EG -_ is the proportion of the EdU positive among the GFP negative cells, N_EG_ is the number of EdU positive GFP positive cells and N_G_ is the number of GFP positive cells. The standard deviation for a binomial distribution in the denominator negatively weights apparent hits based on only a small number of green cells. Wells where N_EG_<50 were rejected from analysis. The advantage of this approach is that the negative control/untransuced cells are in the same well and are exposed to the same micro-environment as the test/transduced cells, so that the score compensates for the variation due to plate-edge effects and inaccuracies in pipetting volume, as well as different transduction rates in the test wells. This method assumes that GFP positive cells express the test protein while the GFP negative cells do not, and requires that both transduced and untransduced cells be present in the well. It can be assumed that GFP positive cells expressed the ORF, because the ORF is located upstream of the IRES-GFP cassette on the same transcript, and it has been extensively documented that proteins 5′ to an IRES are expressed at levels equal to or higher than the proteins downstream of this sequence [52], [53], [54]. Moreover, we have confirmed that GFP expression correlates with expression of the test ORF as detected by immunofluorescence for at least 4 genes (see Figure S4).

As there was considerable variability between plates, z-scores were normalized by plate median centering. Positive control wells and wells containing fewer than 50 GFP positive cells were excluded from median calculations. After the inter-plate variation has been removed, and positive controls excluded, the normalised z-scores were normally distributed (Shapiro test with a p-value>0.05). These normalized z-scores were then used for hit selection by employing Q-Q plots (see text and Figure 3).

Cell cycle analysis of hit plates was performed by rescanning images with the ArrayScan CellCycle.v3 application. Intensity peak thresholds in the DAPI channel were set individually for each plate using untransduced wells to model intensity curves.

## Supporting Information

Figure S1 **Comparison of Plate reader, high-content imaging and flow cytometer analysis of GFP fluorescence following transfection.** 96-well microplates were seeded with HEK293T cells and transfected with lentiviral plasmids as described in methods. A, cells were fixed and stained with DAPI, and the plate scanned on either the FLUOstar Optima Microplate Fluorometer (plate reader) or with Cellomics ArrayScan HCS reader. B, live cells were washed with PBS and scanned on the plate reader. Cells in each well were then trypsinised, collected into 5 ml tubes and fixed. The tubes were individually scanned on a BD FACS Canto Flow Cytometer (FACS).
(TIF)

Figure S2 **Transfection and transduction rate variation between genes and within replicates of the same gene.** A - Transfection rate for vectors expressing the indicated genes was estimated by GFP fluorescence of transfected HEK293T as measured by plate reader. B - Transduction rate was obtained using the HCS reader by scanning the MCF-10A cells exposed to the viral supernatant derived from the HEK293T cells in A. Shaded bars within a group represent replicate wells.
(TIF)

Figure S3 **Regression plots for identifying EdU and GFP positive cells.** A - Scatter-plot of total nuclear DAPI vs total nuclear Cy5 fluorescence intensity. A regression method (see Materials and Methods) fits a linear approximation to the background of Cy5-EdU negative nuclei and identifies outliers, the Cy5-EdU positive cells (shown in red). B - Plots of log_2_(variance GFP intensity ) vs log_2_(mean GFP intensity) identify false GFP-positive cells as a distinct cloud with above-threshold GFP signal but with high fluorescence variance. Identified GFP positive cells are shown in green.
(TIF)

Figure S4 **Comparison of library ORF and GFP expression.** Transduced MCF-10A cells were processed as in the screen assay, except that after fixation they were immunolabelled with primary antibody against the ORF (monoclonal mouse anti-cytokeratin 19 (Invitrogen) or rabbit polyclonal anti-CDK2 (B. G., unpublished), and then secondary antibody conjugated to TRITC (anti-mouse-TRITC or anti-rabbit-TRITC, respectively (Santa-Cruz)). A- Immunofluorescence micrographs of cells overexpressing CMV-driven KRT19 (KRT19-GFP) or CDK2 (CDK2-GFP), followed by the IRES-driven GFP, or empty vector expressing GFP alone. Colocalisation of the GFP and TRITC signal was observed only if the cells were treated with the antibody corresponding to the overexpressed ORF. B – scatter plots of GFP vs TRITC signal intensity obtained by high-content image analysis of the immunolabelled cells, indicating presence of the above background TRITC signal only in cells overexpressing the ORF corresponding to the targeted antigen. Similar data were obtained for cells transduced with CCNE1 and PCNA overexpression clones (not shown).
(TIF)

Table S1 **Primary screen analysis.**
(XLSX)

Table S2 **Confirmation screen analysis.**
(XLSX)

Table S3 **Hit proliferation rate analysis.**
(XLSX)

Table S4 **Hit cell cycle analysis.**
(XLSX)
