# Supplementary figures and images for: A High-Throughput Platform for Lentiviral Overexpression Screening of the Human ORFeome
Source: PLoS One. 2011 May 24;6(5):e20057. doi: 10.1371/journal.pone.0020057 (PMC3101218; doi:10.1371/journal.pone.0020057)

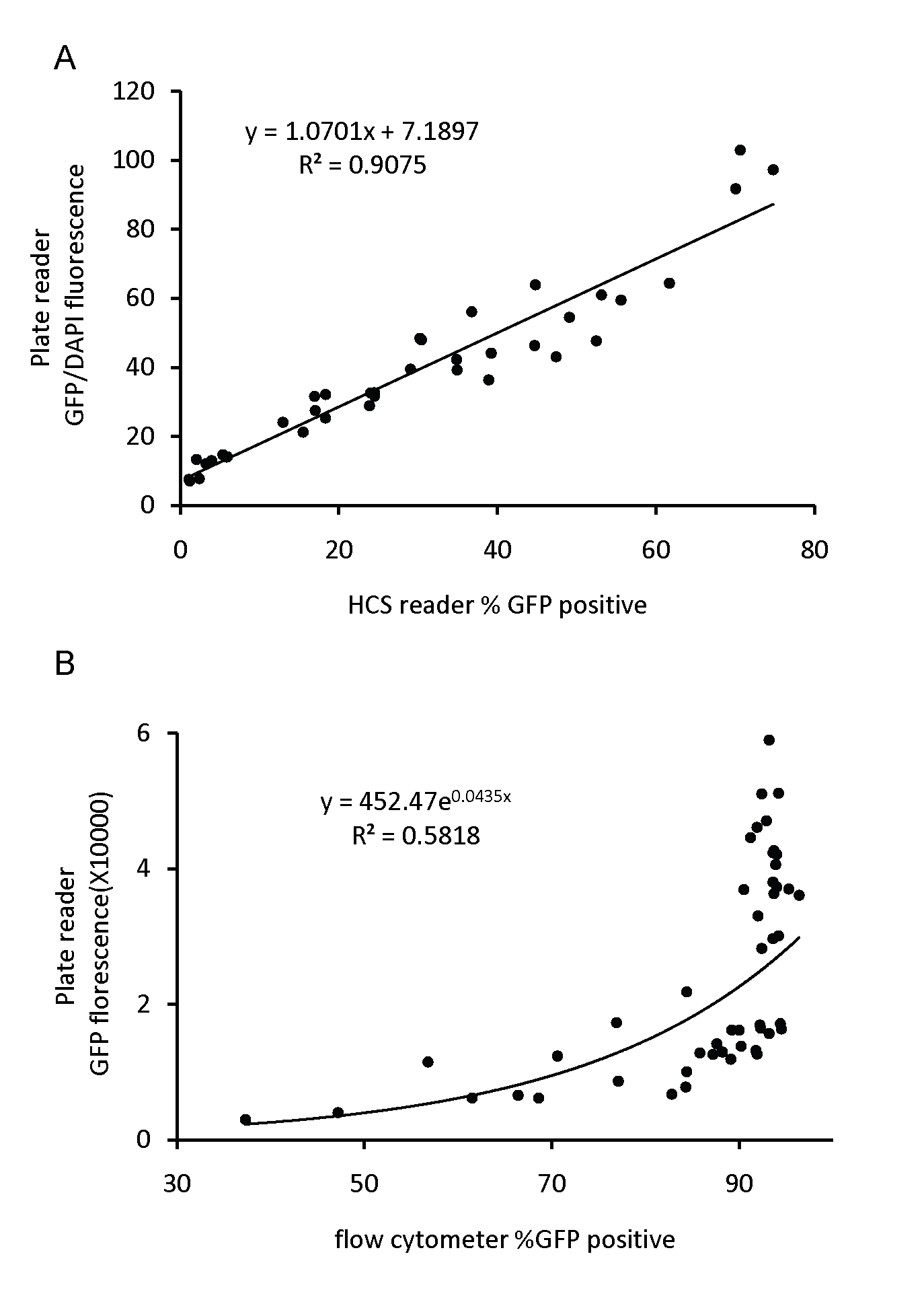

Supplement: Figure S1 — Comparison of Plate reader, high-content imaging and flow cytometer analysis of GFP fluorescence following transfection. 96-well microplates were seeded with HEK293T cells and transfected with lentiviral plasmids as described in methods. A, cells were fixed and stained with DAPI, and the plate scanned on either the FLUOstar Optima Microplate Fluorometer (plate reader) or with Cellomics ArrayScan HCS reader. B, live cells were washed with PBS and scanned on the plate reader. Cells in each well were then trypsinised, collected into 5 ml tubes and fixed. The tubes were individually scanned on a BD FACS Canto Flow Cytometer (FACS). (TIF) [file pone.0020057.s001.tif]

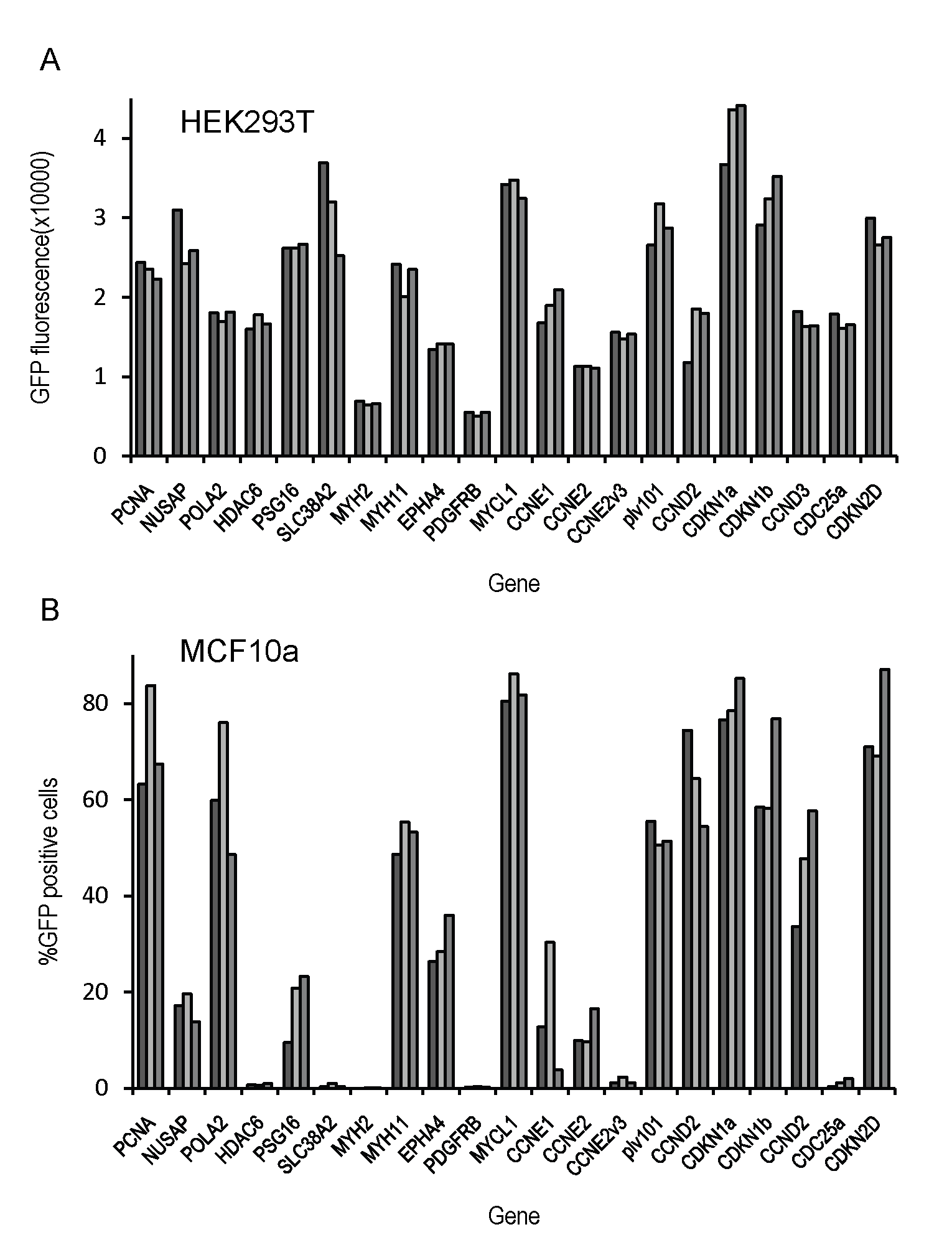

Supplement: Figure S2 — Transfection and transduction rate variation between genes and within replicates of the same gene. A - Transfection rate for vectors expressing the indicated genes was estimated by GFP fluorescence of transfected HEK293T as measured by plate reader. B - Transduction rate was obtained using the HCS reader by scanning the MCF-10A cells exposed to the viral supernatant derived from the HEK293T cells in A. Shaded bars within a group represent replicate wells. (TIF) [file pone.0020057.s002.tif]

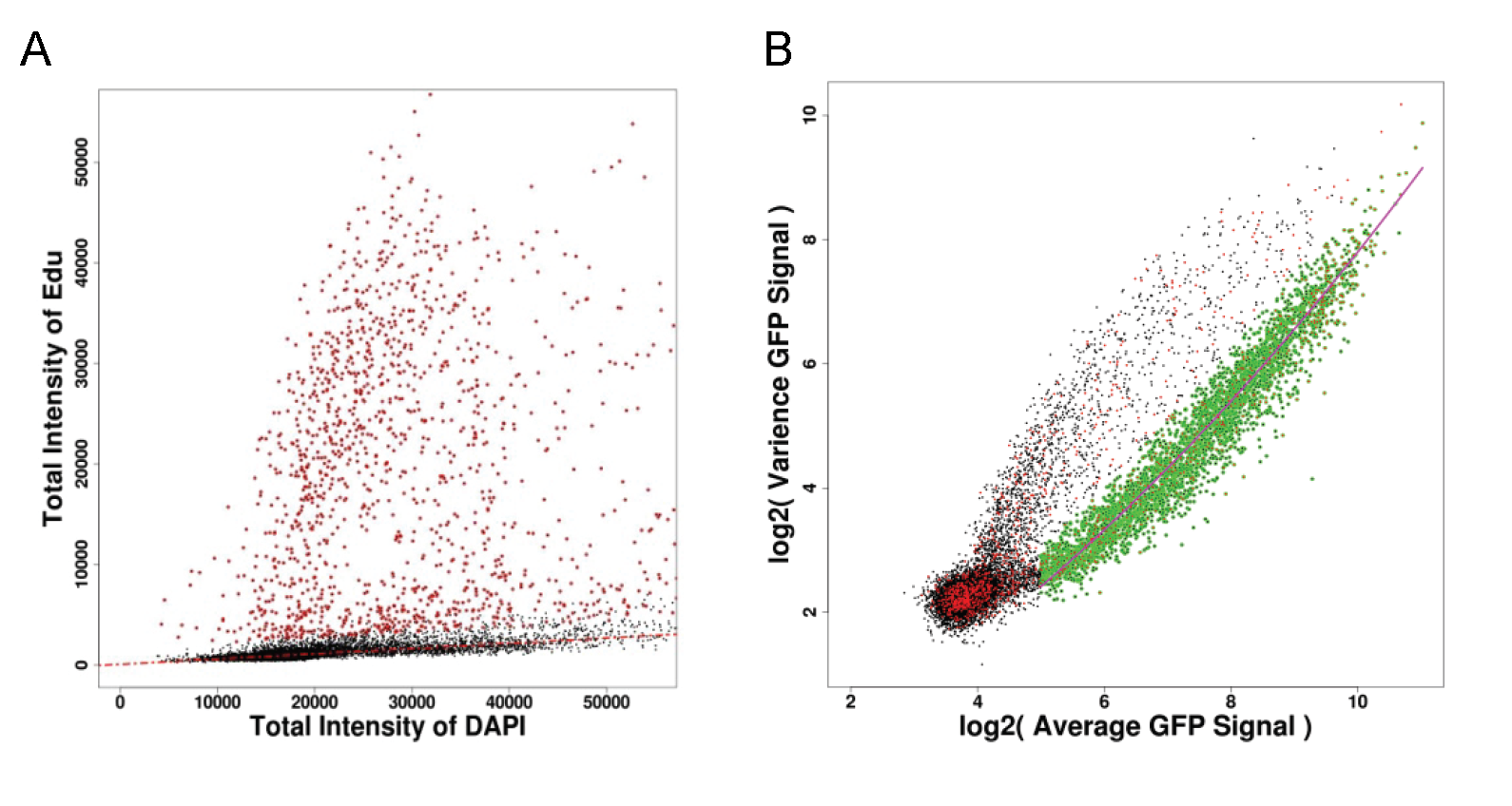

Supplement: Figure S3 — Regression plots for identifying EdU and GFP positive cells. A - Scatter-plot of total nuclear DAPI vs total nuclear Cy5 fluorescence intensity. A regression method (see Materials and Methods) fits a linear approximation to the background of Cy5-EdU negative nuclei and identifies outliers, the Cy5-EdU positive cells (shown in red). B - Plots of log2(variance GFP intensity ) vs log2(mean GFP intensity) identify false GFP-positive cells as a distinct cloud with above-threshold GFP signal but with high fluorescence variance. Identified GFP positive cells are shown in green. (TIF) [file pone.0020057.s003.tif]

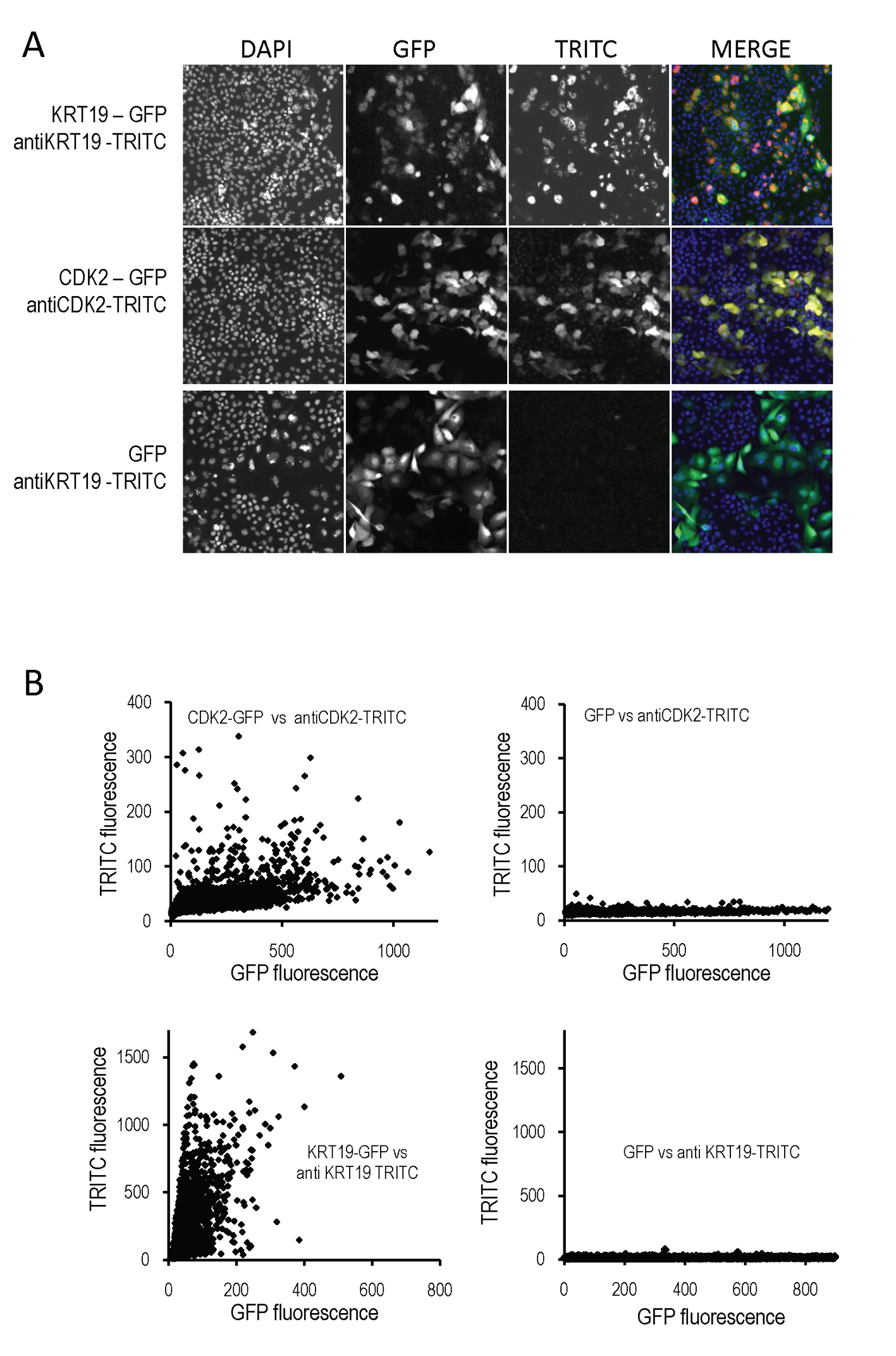

Supplement: Figure S4 — Comparison of library ORF and GFP expression. Transduced MCF-10A cells were processed as in the screen assay, except that after fixation they were immunolabelled with primary antibody against the ORF (monoclonal mouse anti-cytokeratin 19 (Invitrogen) or rabbit polyclonal anti-CDK2 (B. G., unpublished), and then secondary antibody conjugated to TRITC (anti-mouse-TRITC or anti-rabbit-TRITC, respectively (Santa-Cruz)). A- Immunofluorescence micrographs of cells overexpressing CMV-driven KRT19 (KRT19-GFP) or CDK2 (CDK2-GFP), followed by the IRES-driven GFP, or empty vector expressing GFP alone. Colocalisation of the GFP and TRITC signal was observed only if the cells were treated with the antibody corresponding to the overexpressed ORF. B – scatter plots of GFP vs TRITC signal intensity obtained by high-content image analysis of the immunolabelled cells, indicating presence of the above background TRITC signal only in cells overexpressing the ORF corresponding to the targeted antigen. Similar data were obtained for cells transduced with CCNE1 and PCNA overexpression clones (not shown). (TIF) [file pone.0020057.s004.tif]
